# Neurophysiologic monitoring for treatment of upper lumbar disc herniation with percutaneous endoscopic lumbar discectomy: A case report on the significance of an increase in the amplitude of motor evoked potential responses after decompression and literature review

**DOI:** 10.1016/j.ijscr.2020.01.042

**Published:** 2020-02-06

**Authors:** Shenghua He, Zhiqiang Ren, Xiufang Zhang, Jiao Li

**Affiliations:** a Department of Spinal Surgery, Shenzhen Traditional Chinese Medicine Hospital, Shenzhen, 518033, China; b Department of Paediatrics, Shenzhen Hospital of Guangzhou University of Chinese Medicine, Shenzhen, 518034, China

**Keywords:** LDH, Lumbar Disc Herniation, HIVD, Upper Lumbar Herniated Intervertebral Disc, PELD, Percutaneous Endoscopic Lumbar Discectomy, IONM, Intraoperative Neurophysiological Monitoring, MEP, Motor evoked potentials, frEMG, Free running electromyologram, VAS, Visual Analogue Scale, JOA, Japanese Orthopaedic Association, ODI, The Oswestry Disability Index, Intraoperative neurophysiological monitoring, Percutaneous endoscopic lumbar discectomy, Upper lumbar disc herniation, Case report

## Abstract

**Introduction:**

Intraoperative neurophysiological monitoring (IONM) has been widely used in spinal surgery. There is lack of report about IONM for the treatment of upper lumbar disc herniation (HIVD) with percutaneous endoscopic lumbar discectomy (PELD), the relationship between an immediate increase in amplitude of intraoperative MEP after decompression and improvement of the treated levels is rarely studied. Here we reported a surgical case in which an elderly patient with HIVD underwent PELD with IONM and there was a distinct increase in the amplitude of MEP after decompression, showing an immediate improvement of the treated levels.

**Presentation of case:**

A 60-year-old male patient underwent PELD to remove the large disc fragments extruded upward into L2-3 intervertebral space. The nucleus pulposus was successfully removed and an increase in the amplitude of MEP responses after decompression was observed. Pain was alleviated immediately after operation and no complication and recurrence was observed at 1 year follow-up.

**Discussion:**

This is a surgical case of PELD for HIVD with IONM. In this study, we reviewed related studies on PELD for HIVD with IONM and discussed the relationship between an immediate increase in amplitude of intraoperative MEP and the outcomes of the treated level.

**Conclusion:**

We conclude an immediate increase in amplitude of MEP after decompression can be considered as an improvement of the treated levels. IONM can not only be used to prevent the risk of neurological injury in PELD for HIVD, but also be helpful to surgeon to judge the efficacy of decompression via an immediate increase in amplitude of intraoperative MEP.

## Highlights

• There was an increase in the amplitude of MEP after PELD surgery decompression.
• The immediate increase in amplitude of MEP can be considered as an improvement of the treated levels.
• The immediate increase in amplitude of MEP can help surgeon to judge the efficacy of nerve root decompression.

## 1 Introduction

HIVD is defined as L1-2 and L2-3 levels and the prevalence of HIVD is lower (about 1% of all LDH) compared to that of the lower lumbar levels. It was reported [1] that patients with HIVD had significant higher incidence of prior surgery compared to those with lower LDH, the effect of surgery for disc herniation at the upper lumbar levels is less satisfactory and the risk is greater than for those treated at lower lumbar levels. In recent years, PELD has become more and more popular with patients suffering from LDH due to minimal bone and soft tissue damage and rapid postoperative recovery. However, the anatomy of the conusmedullaris and narrow spinal canal at L1-2 and L2-3 levels bring a great risk and technical challenge for surgeons. In order to decrease the risk of neurological injury, IONM is used to provides early detection to the surgeon regarding neural function during surgery.

**Figs. 1 and 2:**
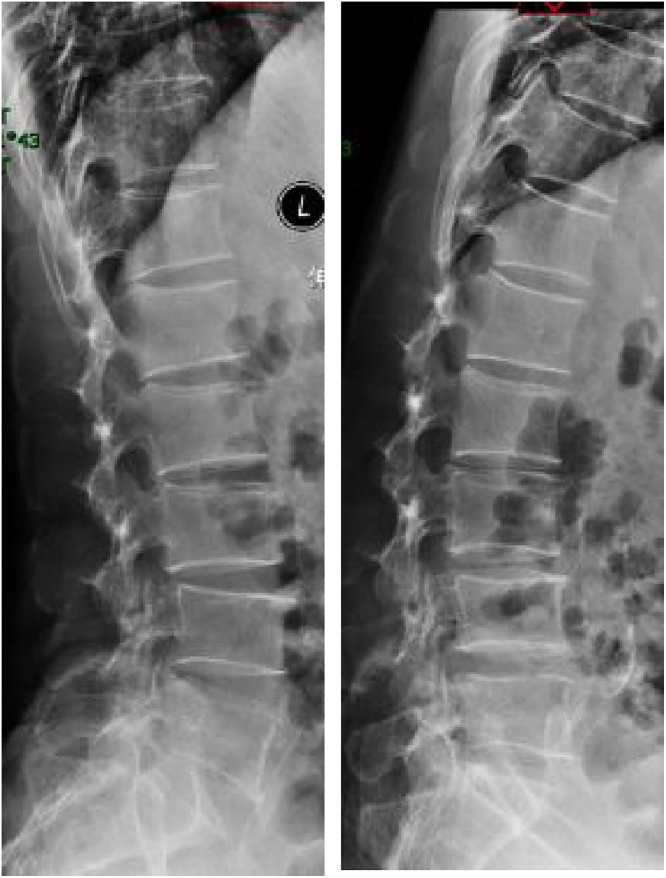
Extension and flexion X-ray showed spinal column unstable was not observed.

Currently, most of our spinal surgeries were monitored by MEP. During operation, we mainly focused on the reduction in amplitude of MEPs. A drop in mMEP of 80% or complete loss from baseline amplitude was considered a significant finding. However, an immediate increase in the amplitude of mMEPs after decompression was observed accidentally. We found intraoperative MEPs improvement after decompression and its relation to clinical outcome of the treated levels have been rarely studied.We report a case of PELD for HIVD at L2-3 level with IONM and the work has been reported in line with the SCARE criteria [2]. With the help of IONM, we successfully get the whole free nucleus pulposus removed without any nerve root injury. What’s more, an immediate increase in amplitude of intraoperative MEP after decompression was detected and an immediate clinical improvement of the treated levels was observed after operation.With this case, we focus on the importance of the relationship between the increase in amplitude of MEP responses after decompression and the efficacy of the treated levels. We put forward this conclusion that an immediate increase in amplitude of intraoperative MEP can be considered as an improvement of the nerve function after decompression and the increase in amplitude of intraoperative MEP can provide an immediate feedback to surgeons to judge the efficacy of nerve root decompression. We expect more cases to be reported to provide further validation of an increase in the amplitude of MEP responses after decompression and its relation to the clinical outcome of PELD surgeries and serve our patients better (Figs. 1 and 2).

## 2 Presentation of case

**Figs. 3 and 5:**
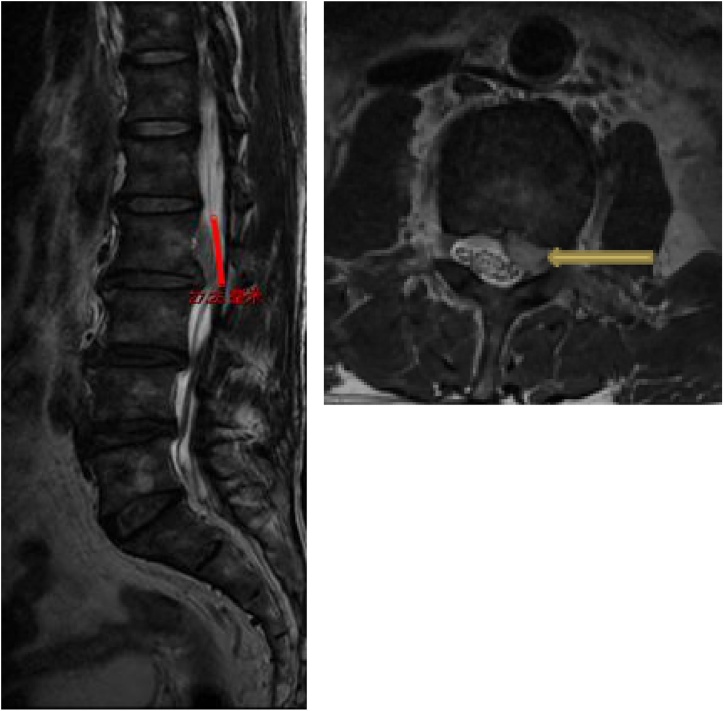
MRI showed that a large disc fragments extruded upward into L2-3 intervertebral space and the left nerve root was compressed.

**Figs. 4 and 6:**
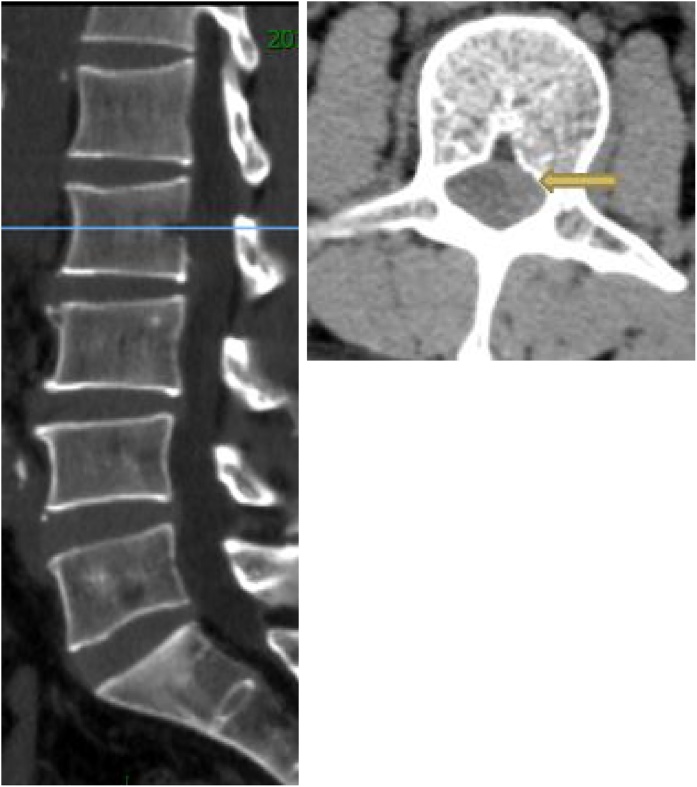
CT scans showed no intervertebral disc calcification was observed.

**Fig. 7:**
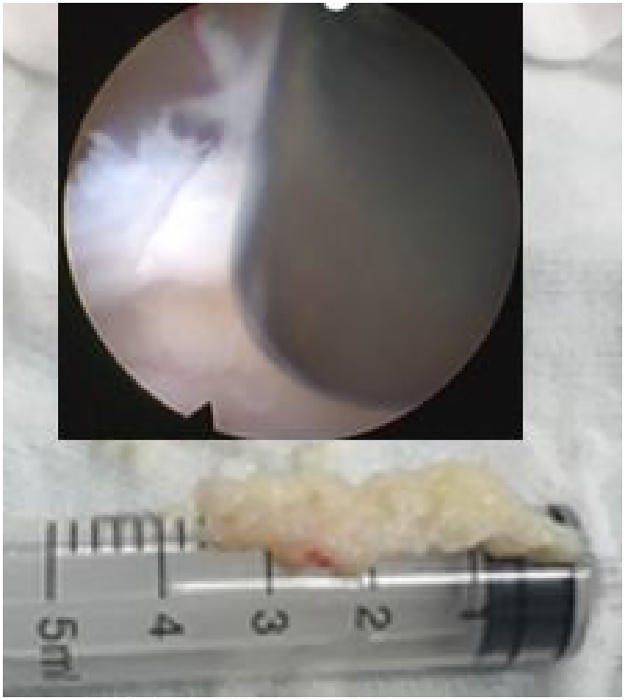
The free nucleus pulposus was completely removed under endoscopy.

**Figs. 8 and 9:**
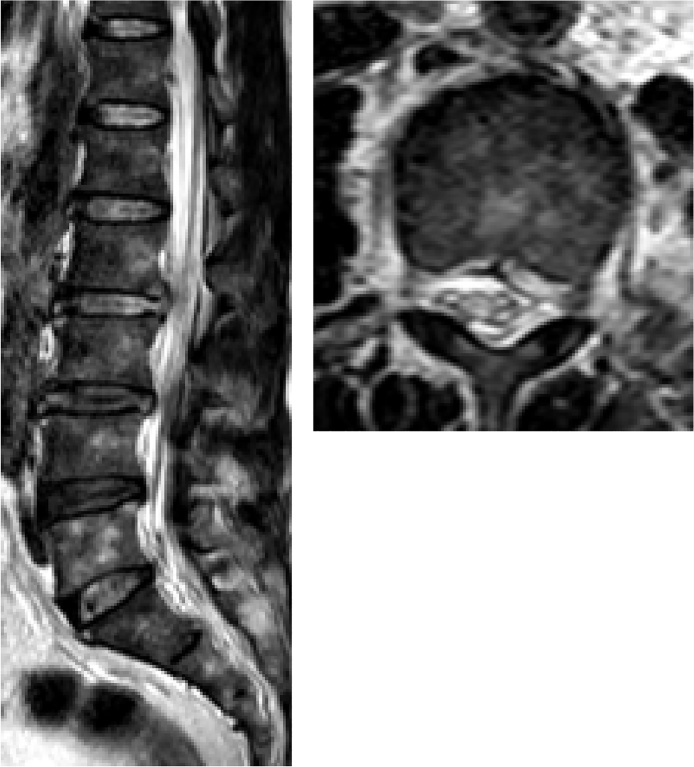
The postoperative MRI demonstrated the disc fragment was removed and left nerve root was decompressed.

**Fig. 10:**
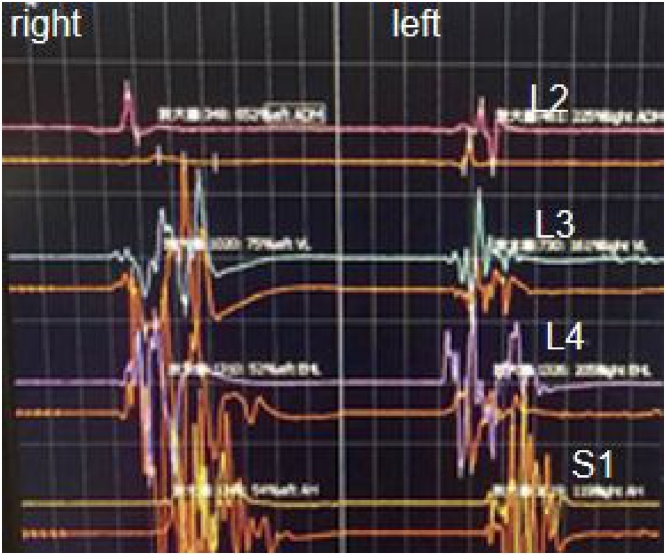
Monitoring showed an immediate increase in amplitudes of MEP response occurred after decompression.

A 60-year-old male patient was admitted to our department, suffering from severe low back pain for more than 10 years and progressive left lower limb radicular pain and numbness in the last 1 week. The VAS of low back and left leg pain was 8. JOA scores were 11 and the ODI score was 64%. Physical examination showed hypoesthesia of left lower extremity. The muscle strength of left quadriceps (front thigh) muscles and first toe was 4/5 grades. The Lasegue sign of left leg was positive with the angle of 30°, and the left knee reflex was weakened. Magnetic resonance imagining and CT revealed that left nerve roots were compressed by herniated disc between L2-3 and a large free nucleus pulposus tissue dissociated upward (Figs. 3 and 5, 4 and 6). After failure of conservative treatment, he underwent PELD surgery with IONM. The surgery was performed with standard surgical procedure under general anesthesia by a senior surgeon. The patient were placed in the prone position on a operating table and neurophysiological potentials were monitored throughout surgery. MEP and frEMG were performed during surgery to assess the function of the spinal cord and provides early detection to prevent latrogenic neurological deficits. MEP and frEMG were recorded bilaterally from the iliopsoas, rectus femoris, tibialis anterior and medial gastrocnemius muscles in the lower extremities using a pair of needle electrodes. No abnormal frEMG signals were found during the operation. Monitoring showed a decrease amplitude of MEP in the compressed nerve root and an immediate increase in amplitudes of MEP response occurred (Fig. 10) after the free nucleus pulposus being removed (Fig. 7), showing an indication of satisfactory efficacy of decompression of the treated levels (Figs. 8 and 9).

Upon patient awakening after completion of the surgery, the patient had pain of low back and leg relieved immediately and the VAS decreased from 8 to 0. He got out of bed and discharged from hospital the next day. One month after the surgery, the VAS of low back and left leg pain was 0. JOA scores were 23 and the ODI score was 24%. One year outpatient follow-up, there was still no pain and numbness was improved greatly.

## 3 Discussion

We began to perform PELD technique with IONM in recent years. So far we have finished 9 cases of PELD for HIVD with IONM. Intraoperative MEPs improvement after decompression was only found in one case of the 9 patients. In this study, we described an elderly patient with a large disc fragments extruded upward into L2-3 intervertebral space not treatable by any conventional treatment. The patient underwent PELD under general anesthesia and was monitored with MEP and frEMG. With the help of IONM, we successfully got the whole free nucleus pulposus removed without nerve injury and the satisfied outcome was confirmed by the immediate increase in the amplitude of MEP responses after decompression. To the authors’ knowledge, there are few articles describing PELD for HIVD with IONM and discussing the relationship between the increase in the amplitude of intraoperative MEP after decompression and clinical outcomes of the treated levels.In this case, we believe that these issues are worthy of discussion.

The herniations of L1-2, L2-3 are less common, accounting for only 5% of total cases of LDH [3]. Due to clinical symptoms and physical examination associated with HIVD are non-specific, it is often difficult to make an accurate diagnosis. What’s more, it was reported that patients with HIVD had significant higher incidence of surgery treatment compared to those with lower LDH and clinical outcome of surgery for HIVD is less satisfactory than for those treated at lower lumbar levels. Sanderson [4] reported that postoperative good or excellent prognosis was only 53% in patients with HIVD.In this case, the patient had nonspecific symptoms, such as anterior thigh pain and localized low back pain and suffered from pain for more than 10 years, but the conservative treatment was not effective, which was in consistent with literature reports. However, the patient’s surgical outcome of the treatment with PELD was fairly satisfactory, which was different from the literature report that clinical outcome of surgery for HIVD is less satisfactory.

It was reported that the choice of surgical method was an important issue when treating patients with HIVD due to the special anatomic structure, such as the narrower spinal canal and the level of the conusmedullaris of the upper lumbar compared with the lower spinal canal [5]. At present, minimally invasive surgical technique [6] have been reported for treating patients with disc herniation in the upper lumbar spine and traditional open surgery was widely considered to lead to spinal instability due to wide laminectomy at the upper lumbar levels.

As minimally invasive surgery (MIS) is becoming more and more popular, PELD has become an alternative treatment due to less invasive to soft tissue and a faster recovery. What’s more, with the development of IONM, it has gradually been utilized in PELD surgery to assess the function of the spinal cord and provides early detection to prevent latrogenic neurological deficits [7]. In order to minimize the surgical trauma and reduce the risk of postoperative neurological complications, we performed PELD surgery for the patient and IONM was monitored throughout surgery. The patient had pain of low back and leg relieved immediately after operation and got out of bed and discharged from hospital the next day. The surgical outcomes demonstrated that HIVD can be treated by PELD operation with less wound, quicker recovery and lower risk of postoperative complications.

IONM has long been utilized during spinal deformity surgery. Lots of previous studies have reported that combined mMEP, SEP and frEGM monitoring provides a large number of essential information for early detection to reduce postoperative sensory and motor deficits in spinal cord surgery [8]. In our study, frEMG and MEP were monitored throughout surgery. When an abnormality of MEP or frEMG was detected, surgeon and anesthesiologist were immediately informed. If significant changes occurred, the surgeon was informed and asked to stop the current surgical intervention. If the changes returned to normal, operative activities were resumed. According to the related reports in the literature, neurotonic discharges of frEMG activity were associated with nerve root irritation, which can help surgeon to take surgical action to relieve the irritation [9]. In this case, there was no abnormal changes of frEMG activity being detected during the surgery. Although, many published reports had demonstrated that decrease in amplitude of MEP were associated with new nerve root deficits in surgical cases [10], there were rare reports on the increase in amplitude of MEP and its relation of decompression. We reviewed a great deal of reports published in the past years and found only one article that described that an improvement of 30% in MEP responses was observed after discectomy and the author believed that these findings suggested an immediate improvement of the treated levels [11]. In this study, we observed the same results that a low amplitude of MEP responses was observed before decompression but an remarkable increase in the amplitude of MEP responses occurred after decompression. It was verified that when the patient waked up, the pain disappeared immediately, there was no recurrence of pain during 1 year follow-up. We believed these findings suggested that the increase of amplitude of MEP might be related to the outcome of decompression, showing an immediate improvement of the treated levels. We are aware of the number of patients was small to this study and we believe the study indicated that more studies being needed to verify this clinical question.

## 4 Conclusion

In this case report, it is demonstrated that IONM can be used to decrease the risk of neurological injury in PELD for HIVD. What’s more, an immediate increase in amplitude of intraoperative MEP after decompression was detected, showing an immediate improvement of the treated levels. We put forward this conclusion that an immediate increase in amplitude of intraoperative MEP can be considered as an improvement of the nerve function after decompression and the increase in amplitude of intraoperative MEP can provide an immediate feedback to surgeons to judge the efficacy of nerve root decompression.To the authors’ knowledge, the increase in amplitude of intraoperative MEP and its relation to early and long-term postoperative clinical outcome have been incompletely studied and we believe the study indicated that more studies being needed to address this clinical question.

## Conflicts of interest

The authors report no conflict of interest.

## Funding

No funding was received for this study.

## Ethical approval

Not applicable; this is a case report and literature review. All patient identifying details are omitted.

## Consent

Written informed consent was obtained from the patient for publication of this case report and accompanying images. All patient identifying details are omitted.

## Author contribution

Shenghua He: study concept, data collection and analysis, data interpretation. Zhiqiang Ren: study concept, writing the manuscript, literature review. Xiufang Zhang: study concept, data interpretation. Jiao Li: data collection and analysis.

## Registration of research studies

Not applicable; this is a case report and literature review. All patient identifying details are omitted.

## Guarantor

Shenghua He and Zhiqiang Ren.

## Provenance and peer review

Not commissioned, externally peer-reviewed.

## References

[1] Saberi H., Isfahani A.V. (2008). Higher preoperative Oswestry Disability Index is associated with better surgical outcome in upper lumbar disc herniation. Eur. Spine J..

[2] Agha R.A., Borrelli M.R., Farwana R., Koshy K., Fowler A., Orgill D.P., For the SCARE group (2018). The SCARE 2018 statement: updating consensus surgical CAse REport (SCARE) guidelines. Int. J. Surg..

[3] Albert T.J., Balderston R.A., Heller J.G., Herkowitz H.N., Garfin S.R., Tomany K. (1993). Upper lumbar disc herniations. J. Spinal Disord..

[4] Sanderson S.P., Houten J., Errico T., Forshaw D., Bauman J., Cooper P.R. (2004). The unique characteristics of “upper” lumbar disc herniation. Neurosurgery.

[5] Choi J.W., Lee J.K., Moon K.S., Hur H., Kim Y.S., Kim S.H. (2007). Transdural approach for calcified central disc herniation of the upper lumbar spine. Technical note. J. Neurosurg. Spine.

[6] Takagi Y., Yamada H., Ebara H., Hayashi H., Kidani S., Toyooka K., Kitano Y., Kagechika K., Tsuchiya H. (2018). Recurrent upper lumbar disc herniation treated via the transforaminal approach using microendoscopy-assisted lumbar discectomy: a case report. J. Med. Case Rep..

[7] Alemo S., Sayadipour A. (2010). Role of intraoperative neurophysiologic monitoring in lumbosacral spine fusion and instrumentation: a retrospective study. World Neurosurg..

[8] Raynor B.L., Bright J.D., Lenke L.G., Rahman R.K., Bridwell K.H., Riew K.D. (2013). Signifificant change or loss of intraoperative monitoring data: a 25-year experience in 12,375 spinal surgeries. Spine.

[9] Bose B., Wierzbowski L.R., Sestokas A.K. (2002). Neurophysiologic monitoring of spinal nerve root function during instrumented posterior lumbar spine surgery. Spine.

[10] Duncan J.W., Bailey R.A., Baena R. (2012). Intraoperative decrease in amplitude of somatosensory-evoked potentials of the lower extremities with interbody fusion cage placement during lumbar fusion surgery. Spine.

[11] Reis Rodrigues L.M., da Rosa F.W.F., Rodriguez Ferreira R.J., Ueno F., Milani C. (2011). Herniated lumbar disc surgery in triathlon athletes with intraoperative neurophysiologic monitoring. Einstein.

